# *MAPT* Subhaplotypes in Different Progressive Supranuclear Palsy Phenotypes

**DOI:** 10.3390/biomedicines13061405

**Published:** 2025-06-07

**Authors:** Monica Gagliardi, Radha Procopio, Alessia Felicetti, Grazia Annesi, Mariagrazia Talarico, Basilio Vescio, Aldo Quattrone, Andrea Quattrone

**Affiliations:** 1 Neuroscience Research Center, Magna Graecia University, 88100 Catanzaro, Italy; monica.gagliardi@unicz.it (M.G.); quattrone@unicz.it (A.Q.); an.quattrone@unicz.it (A.Q.); 2 Institute of Neurology, Department of Medical and Surgical Sciences, Magna Graecia University, 88100 Catanzaro, Italy; alessia.felicetti@studenti.unicz.it; 3 Institute for Biomedical Research and Innovation, National Research Council, 87050 Cosenza, Italy; grazia.annesi@cnr.it; 4 Stem Cell Laboratory, Department of Medical and Surgical Sciences, Magna Graecia University, 88100 Catanzaro, Italy; m.talarico@unicz.it; 5 Biotecnomed S.c.a.r.l., 88100 Catanzaro, Italy; b.vescio@unicz.it; 6 Institute of Bioimaging and Complex Biological Systems – National Research Council (IBSBC-CNR), 88100 Catanzaro, Italy

**Keywords:** *MAPT* gene, progressive supranuclear palsy, haplotypes

## Abstract

**Background:** Progressive Supranuclear Palsy (PSP) is a rare neurodegenerative disorder characterized by abnormal tau protein aggregation. The *MAPT* gene encodes for tau protein. The *MAPT* locus harbors two major haplotypes, H1 and H2, with H1 and its subhaplotypes being associated with an increased risk of PSP. **Methods:** In this study, we genotyped rs8070723 in a cohort of 73 PSP patients, including 47 PSP Richardson Syndrome (PSP-RS) and 27 PSP variants (vPSP), and 93 age-matched healthy controls (HC) from Southern Italy. **Results:** Haplotype analysis identified H1 and H2 haplotypes that conferred a risk (OR, 2.620; 95% CI, 1.399–5.140; *p* = 0.0035) and a protective effect (OR, 0.370; 95% CI, 0.196–0.695; *p* = 0.0015), respectively. In addition, we genotyped five *MAPT* variants (rs1467967, rs242557, rs3785883, rs2471738, and rs7521) that, together with rs8070723, defined H1 subhaplotypes. We identified 18 distinct *MAPT* H1 subhaplotypes, among which H1j displayed a nominally significant reduced risk of PSP (OR, 0.201; 95% CI, 0.044–0.915; *p* = 0.0265). **Conclusions:** These findings reinforce the role of *MAPT* genetic variation in PSP pathogenesis and highlight the potential impact of haplotype diversity on disease susceptibility.

## 1. Introduction

Progressive supranuclear palsy (PSP) is a rare neurodegenerative movement disorder with an estimated annual prevalence of 5.8–6.5 cases per 100,000 [1]. PSP is clinically heterogeneous and may manifest various phenotypes, in which the most common form is Richardson Syndrome (PSP-RS), with vertical supranuclear gaze palsy, postural instability, and motor and cognitive dysfunction [2,3]. Other recognized forms (termed PSP variant, vPSP) are PSP with predominant parkinsonism (PSP-P), PSP with progressive gait freezing (PSP-PGF), PSP with predominant corticobasal syndrome (PSP-CBS), PSP with a predominant speech/language disorder (PSP-SL), PSP with predominant frontal presentation (PSP-F), PSP with cerebellar ataxia (PSP-C), and oculomotor dysfunction (PSP-OM) [2,3].

Neuropathologically, PSP is characterized by abnormal accumulation of tau protein in the form of globose neurofibrillary tangles (NFTs), tufted astrocytes (TA), oligodendroglial coiled bodies (CB) and neuropil threads (NT) in the basal ganglia, brainstem, cortical regions, and cerebellum [4].

Tau, a microtubule-associated protein predominantly found in neuronal axons, plays a crucial role in regulating microtubule dynamics, modulating synaptic plasticity and supporting axonal transport [5]. It is organized into four main functional domains: a N-terminal region (NTR), a proline-rich domain (PRR), a microtubule-binding domain (MTBD), and the C-terminal assembly region [6].

Tau protein is encoded by *MAPT*, a long gene of 16 exons located on chromosome 17q2 [7]. In the adult human brain, six major tau protein isoforms are generated by alternative splicing of exons 2, 3, and 10, which determine the number of N-terminal inserts (0N, 1N, or 2N) and C-terminal repeat domains (3R or 4R) [8]. Alternative splicing of exons 2 and 3 generates isoforms 0N if both 2 and 3 exons are absent, 1N if only exon 2 is included, and 2N if both exons are included. The exclusion or inclusion of exon 10 determines whether an isoform has three (3R) or four (4R) repeat domains, respectively [6,9]. The ratio of 4R to 3R is approximately equal (1:1); however, an increased 4R:3R ratio has been linked to PSP-RS pathology. This imbalance enhances tau phosphorylation and aggregation, reduces its binding affinity to microtubules, and consequently impairs normal microtubule function, contributing to neurodegeneration [10].

*MAPT* is located in a region of chromosome 17 with extensive linkage disequilibrium (LD) that has undergone a 900 kb ancestral inversion, giving rise to two major haplotypes, H1 and H2 [11]. These haplotypes differ in orientation and do not recombine. While the H1 haplotype is widespread across all populations, the H2 haplotype is primarily found in Middle Eastern and European populations (~25%), but it is rare in Central Asia and nearly absent in African, East Asian, and Native American populations [12,13]. H1 haplotype, previously described as a risk factor for PSP, can be further divided into more than 20 subhaplotypes [14,15]. Among these, H1c, H1d, H1g, and H1o have consistently been associated with an increased risk of PSP [13,16,17,18]. In contrast, H2 haplotype appears to have a protective role [19,20,21].

Beyond the haplotypic architecture, several single nucleotide polymorphisms (SNPs) within *MAPT* have been implicated in modulating disease susceptibility and tau-related pathophysiology. For instance, the A allele of rs242557 has been linked to increased plasma tau levels and enhanced transcriptional activity of the *MAPT* promoter, indicating a regulatory role in gene expression [22] Similarly, rs3785883 has been associated with higher cerebrospinal fluid (CSF) tau concentrations and accelerated functional decline in patients with Alzheimer’s disease, suggesting a broader role in tau-related neurodegeneration [23]. The T allele of rs2471738 has been correlated with elevated *MAPT* expression in the human hippocampus, supporting its involvement in tissue-specific gene regulation [24]. Notably, rs8070723 is in strong LD with rs242561, which resides within an antioxidant response element (ARE) of *MAPT* that binds NRF2/sMAF transcription factors. This genomic context implies that rs8070723 may influence the transcriptional regulation of *MAPT* in response to oxidative stress, thereby modulating tau expression [25]. Additionally, rs1467967 has been associated with increased levels of total tau (t-tau) and phosphorylated tau (p-tau181) in the CSF, particularly among individuals carrying the AA or AG genotypes [26]. Lastly, rs7521 has been shown to affect the expression of the 4R tau isoform, particularly in carriers of the H1c haplotype—an isoform that predominates in PSP and other tauopathies [27].

In this study, we performed for the first time a genetic association analysis to investigate the link between *MAPT* haplotypes and PSP susceptibility evaluated in the whole PSP group and separately in the different phenotypes (PSP-RS and vPSP). We analyzed six *MAPT* SNPs in a cohort of 73 PSP patients and 93 age-matched healthy controls from Southern Italy.

## 2. Materials and Methods

### 2.1. Patients

We enrolled unrelated 73 PSP cases (46 RS and 27 Variant) and HC subjects (*n* = 93) at the Neuroscience Research Center, University Magna Graecia of Catanzaro, Italy. All of them were Caucasian, originated in Calabria, Southern Italy. The two groups were age matched, with PSP cases having an average age at blood collection of 69.90 (65.75% male) while healthy controls of 72.85 (43.01% male). Demographic information of the patient and control groups is summarized in Table 1. The study was conducted in accordance with the Declaration of Helsinki and approved by the Calabria Region Ethics Committee under protocol code 143 on 13 May 2024. All participants provided informed consent after receiving a detailed explanation of the study’s purpose.

### 2.2. Genetic Analysis

Genomic DNA was extracted from peripheral blood samples of both PSP patients and healthy controls using Maxwell^®^ RSC Blood DNA kit. Genotyping was conducted in triplicate using TaqMan SNP genotyping assays on QuantStudio 5 instrument (Applied Biosystems, Waltham, MA, USA), adhering to the manufacturer’s instructions. Genotype calling was performed using QuantStudio^TM^ Design & Analysis software v.1.5.3 (Applied Biosystems) to ensure accurate variant identification. Genotyping of six *MAPT* variants (rs1467967, rs242557, rs3785883, rs2471738, rs8070723, and rs7521) was performed to evaluate the most common *MAPT* haplotypes [13,14,28,29,30]. Among them, the *MAPT* H2 haplotype was tagged by the variant rs8070723, which was genotyped in all patients and controls. The minor allele G of rs8070723 corresponds to the *MAPT* H2 haplotype, while the major allele A is associated with the *MAPT* H1 haplotype [31]. The rate of genotype calls was 100% in each population. A total of 19 distinct *MAPT* haplotypes, each observed in at least 1% of individuals across the association analyses conducted in this study, are presented in Table 2.

### 2.3. Statistical Analysis

Statistical assessment for the allele and genotype frequencies and Hardy–Weinberg equilibrium (HWE) was made using TaqMan^TM^ Genotyper Software v1.7 (Applied Biosystems). For each SNP, the allele and genotype distributions in PSP cases were compared with those of the HC group. Pairwise LD across *MAPT* for each SNP was then evaluated using both D’ and the squared correlation coefficient (r^2^) with Haploview v.4.2 (Broad Institute, Cambridge, MA, USA). The Expectation-Maximization (EM) algorithm, adjusted for sex, was employed to infer haplotypes and estimate their frequencies from genotype data. Odds ratios (ORs) and 95% confidence intervals (CIs) of inferred haplotypes were calculated using the epi.2by2 function from the epiR package, while odds ratios and CIs of A and G alleles on the rs8070723 SNP were evaluated using logistic regression, adjusting for age and sex as covariates. ORs and CIs were computed in the R statistical computing environment, version 4.2.2 (The R Foundation for Statistical Computing, Vienna, Austria) to assess the association between six variant *MAPT* haplotypes and risk of PSP. Specifically, association tests were conducted for 18 or 19 haplotypes, depending on the patient group under analysis. Comparisons were performed between the following groups: PSP versus HC, PSP-RS versus HC, vPSP versus HC. A Bonferroni correction for multiple testing established statistical significance at *p* ≤ 0.0026 (0.05/19) or *p* ≤ 0.0027 (0.05/18).

## 3. Results

### 3.1. Linkage Disequilibrium Analysis

No significant differences in allele and genotype frequencies were found between PSP and controls (Table 3). None of the six SNPs departed from HWE in both populations (Table 3).

Our analysis of LD patterns at the *MAPT* locus supports the presence of two distinct haplotype lineages, H1 and H2, which have remained evolutionarily separate for approximately 3 million years [11]. By examining D’ and r^2^ in HC and PSP cases, we identified SNP pairs (rs1467967-rs8070723, rs242557-rs8070723, rs3785883-rs8070723, rs2471738-rs8070723, rs8070723-rs7521) that exhibit high LD in both groups, suggesting that they define stable haplotype structures (H1 and H2) rather than random associations (Table 4). Furthermore, the absence of recombination between H1 and H2 supports the use of H2-specific SNP alleles (rs8070723) as reliable surrogate markers for inversion status. This finding has important implications for genetic association studies, as it ensures that the differentiation between H1 and H2 haplotypes can be accurately captured without requiring direct structural analysis.

### 3.2. Association of MAPT rs8070723 Alleles with PSP

In our association analysis, the *MAPT* rs8070723 H2 allele was significantly associated with a reduced risk of PSP (OR, 0.382; 95% CI, 0.195–0.715; *p* = 0.0035) and PSP-RS subtype (OR, 0.250; 95% CI, 0.098–0.556; *p* = 0.0015), with minor allele frequencies of 11.0% in the 73 PSP, and 8.7% in the 46 PSP-RS patient subgroup, compared to 24.7% in the 93 HC. Conversely, the rs8070723 H1 allele was significantly associated with an increased risk of PSP (OR, 2.620; 95% CI, 1.399–5.140; *p* = 0.0035) and PSP-RS (OR, 4.004; 95% CI, 1.798–10.237; *p* = 0.0015), with major allele frequencies of 89.0% and 91.3% in PSP cases and PSP-RS patient subgroup, respectively, compared to 75.3% in HC (Table 5 and Table S1).

### 3.3. Genetic Association of MAPT Haplotypes with PSP

A subhaplotype (H1j) was associated with a nominally significant (*p* < 0.05) decreased risk of PSP in our cohort (H1j: OR, 0.201; 95% CI, 0.044–0.915; *p* = 0.0265) (Table 6 and Table S2), whereas another study reported a significant association with increased risk in a different PSP cohort [14].

Consistently with previously findings [14,20], H2 haplotype showed a significant protective effect on PSP risk (OR, 0.370; 95% CI, 0.196–0.695; *p* = 0.0015) since it was overrepresented in healthy controls (Table 6). Despite the small sample size, H2 frequency was high in both PSP and HC groups as expected in the European population [12].

### 3.4. Haplotype-Based Genetic Stratification of PSP-RS and vPSP

A similar analysis was conducted in two different PSP phenotypes: PSP-RS and vPSP, compared to the HC group. A total of 18 haplotypes (H2 haplotype and 17 H1 subhaplotypes) were found in the PSP-RS/HC group (Table S3), but only H2 had a significant protective effect on the risk of PSP-RS (OR, 0.274; 95% CI, 0.118–0.636; *p* = 0.0014) (Table 7 and Table S4). In the vPSP/HC group, 18 haplotypes (H2 haplotype, one H2 subhaplotype and 16 H1 subhaplotypes) were found (Table S5), but no significant effects were observed (Table 8 and Table S6).

**Figure 1:**
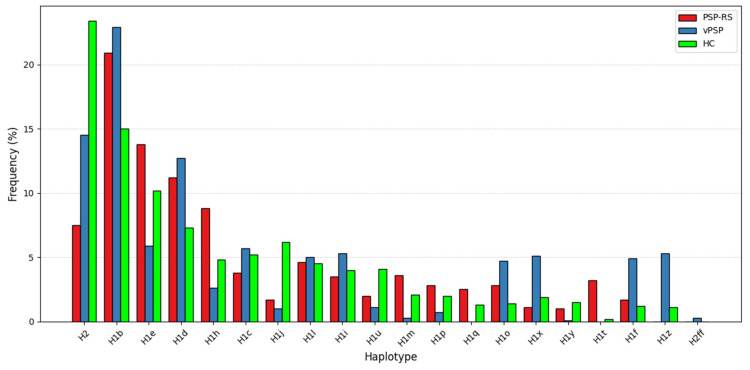
Distribution of haplotype frequencies. The bar plot shows the distribution of *MAPT* haplotypes across PSP-RS, vPSP, and HC groups. Several H1 subhaplotypes, such as H1e, H1q, H1t, H1z, show different distribution in PSP-RS and vPSP, while the H2 haplotype shows a markedly higher frequency in the HC group, supporting its potential protective role.

When stratified by phenotype (PSP-RS and vPSP), the protective trend of H1j haplotype persisted but did not achieve statistical significance, likely due to limited sample sizes. Distribution of haplotype frequencies across the three groups is illustrated in the bar plot (Figure 1), highlighting differences in the prevalence of specific subhaplotypes.

## 4. Discussion

In this study, the association between *MAPT* haplotypes and the risk of PSP was investigated for the first time in a cohort of Italian PSP patients with two different phenotypes (RS and vPSP). Our findings confirm the strong LD at the *MAPT* locus, driven by the 900-kb inversion at 17q21.31, which defines the H1 and H2 haplotypes [11,14]. The absence of recombination between these haplotypes preserves their distinct genetic architecture. Notably, rs8070723 tags the H2 haplotype, enabling efficient haplotype differentiation without structural analysis. This reinforces the evolutionary stability of the *MAPT* inversion and its relevance for genetic association studies.

The analysis of major allele distribution in the rs8070723 SNP among different groups confirmed the association between H1 and the risk of PSP and, in particular, PSP-RS, while the overrepresentation of minor allele in HC confirmed the protective effect of H2 [13,18,28,29].

In our haplotype association analysis, the H1j haplotype, previously identified [14] only in a UK population as being more prevalent in controls than in PSP cases, showed a nominally significant association with a reduced risk of PSP (OR, 0.201; 95% CI, 0.044–0.915; *p* = 0.0265). Although the *p*-value does not reach significance after Bonferroni correction due to sample size limitations, the confidence intervals fall below 1, suggesting a potential protective effect. This trend is further supported by a similarly low frequency in cases compared to controls, even when analyzing PSP subgroups. The observed discrepancy with the UK findings may reflect differences in population-specific genetic backgrounds (Southern Italian vs. British), LD structures, or methodological aspects of cohort design. Nevertheless, additional studies are warranted to better clarify and validate this potential association.

The H1 haplotype has been linked to increased expression of 4-repeat (4R) tau isoforms, which are predominant in PSP pathology, while the H2 haplotype is associated with reduced *MAPT* expression and a lower 4R/3R tau ratio [10]. The protective effects of H2 and H1j may stem from their association with decreased 4R-tau expression, potentially mitigating tau aggregation and neurodegeneration. These functional differences may help explain the observed associations with PSP risk and support further studies to clarify the molecular mechanisms involved.

Our study also examined the distribution of *MAPT* haplotypes in different PSP subtypes, including PSP-RS and vPSP patients. We identified 18 haplotypes in this stratified analysis, but only H2 haplotype reached statistical significance in the PSP-RS/HC after multiple testing corrections, and it appeared to confer greater protective effects in PSP-RS compared to vPSP. Although these associations did not reach statistical significance probably because of small sample size, our analysis revealed distinct H1 subhaplotype distributions between PSP-RS and vPSP subtypes and HC subjects. The exclusive presence of H1t and H1q in PSP-RS and H1z in vPSP suggests that these haplotypes may contribute to different genetic background in PSP subtypes. The observed overrepresentation of subhaplotype H1e in PSP-RS patients, compared to those with vPSP (Figure 1), may reflect underlying genetic factors that contribute to the differentiation of these clinical subtypes. However, future studies on larger international cohorts, including on the whole PSP spectrum and specifically looking at distinct PSP phenotypes, are needed to validate our findings and shed further light on this point.

Despite our study providing valuable insights into *MAPT* haplotypes in PSP, several limitations should be acknowledged. First, our sample size, though comparable to other genetic studies of PSP, remains relatively small, potentially limiting statistical power. Second, our cohort consists exclusively of individuals of Southern Italian ancestry, which may restrict the generalizability of our findings to other populations. Future studies in larger patient cohorts should include diverse ethnic groups to explore potential population-specific effects.

In conclusion, our results reinforce the role of *MAPT* haplotypes in PSP susceptibility and highlight the need for further research to validate and expand upon these findings. Understanding the role of haplotypes in influencing the risk of PSP is a crucial step toward unrevealing the complex genetic underpinnings of this neurodegenerative disorder.

## Figures and Tables

**Table 1:** Age and sex characteristics of the PSP and HC populations. The sample mean ± standard deviation is given for age at blood collection of PSP and HC. Number and percentage of females and males are given for both populations.

	PSP	HC
Mean Age, Range	69.90 ± 6.97	72.85 ± 7.32
Sex, No. (%)		
Female	25 (34.25)	53 (56.99)
Male	48 (65.75)	40 (43.01)

**Table 2:** *MAPT* H2 haplotype and H1 subhaplotypes that were observed in 1% or more of PSP and HC populations in any of the 19 association analyses.

Haplotype	*MAPT* Variant					
	rs1467967	rs242557	rs3785883	rs2471738	rs8070723	rs7521
H1b	G	G	G	C	A	A
H2	A	G	G	C	G	G
H1e	A	G	G	C	A	A
H1d	A	A	G	C	A	A
H1h	A	G	A	C	A	A
H1c	A	A	G	T	A	G
H1l	A	G	A	C	A	G
H1j	A	G	G	C	A	G
H1i	G	A	G	C	A	A
H1u	A	A	G	C	A	G
H1m	G	A	G	C	A	G
H1o	A	A	A	C	A	A
H1x	G	A	A	T	A	G
H1f	G	G	A	C	A	A
H1z	G	A	G	T	A	G
H1p	G	G	G	T	A	G
H1q	A	A	G	T	A	A
H1y [32]	G	A	A	C	A	G
H1t	A	G	A	T	A	G

**Table 3:** Allele and genotype distributions. The calculated HWE *p*-value is the probability of the differences in observed and expected genotype calls accounted for by chance alone. No deviations of the genotype frequencies from HWE equilibrium were detected in either cases or controls.

	**rs1467967**						**rs242557**					
**Sample**	**A**	**G**	**A/A**	**A/G**	**G/G**	**HWE** ***p-*** **Value**	**G**	**A**	**G/G**	**G/A**	**A/A**	**HWE** ***p-*** **Value**
HC	69.9%	30.1%	51.6%	36.6%	11.8%	0.205	68.8%	31.2%	47.3%	43.0%	9.7%	1.000
PSP	58.9%	41.1%	32.9%	52.1%	15.1%	0.520	64.4%	35.6%	41.1%	46.6%	12.3%	0.893
	**rs3785883**						**rs2471738**					
**Sample**	**A**	**G**	**A/A**	**A/G**	**G/G**	**HWE** ***p*** **-Value**	**C**	**T**	**C/C**	**C/T**	**T/T**	**HWE** ***p*** **-Value**
HC	16.1%	83.9%	1.1%	30.1%	68.8%	0.277	87.6%	12.4%	76.4%	22.6%	1.1%	0.686
PSP	24.0%	76.0%	6.8%	34.2%	58.9%	0.605	83.6%	16.4%	68.5%	30.1%	1.4%	0.407
	**rs8070723**						**rs7521**					
**Sample**	**A**	**G**	**A/A**	**A/G**	**G/G**	**HWE** ***p*** **-value**	**A**	**G**	**A/A**	**A/G**	**G/G**	**HWE** ***p*** **-Value**
HC	75.3%	24.7%	58.1%	34.4%	7.5%	0.465	45.2%	54.8%	20.4%	49.5%	30.1%	1.000
PSP	89.0%	11.0%	80.8%	16.4%	2.7%	0.178	63.7%	36.3%	41.1%	45.5%	13.7%	0.605

**Table 4:** Pairwise LD analysis. LD was calculated as D’ and r^2^. A D’ of 0.0 indicates that the markers are assorting independently, while a D’ of 1.0 implies that the less common allele is always found paired with a specific allele at the other marker. r^2^ = 1.0 only when the marker loci also have identical allele frequencies.

SNP1	SNP2	HC		PSP	
		D’	r^2^	D’	r^2^
rs1467967	rs242557	0.102	0.01	0.116	0.005
rs1467967	rs3785883	0.127	0.01	0.352	0.027
rs1467967	rs2471738	0.183	0.011	0.249	0.017
rs1467967	rs8070723	1.0	0.142	0.783	0.053
rs1467967	rs7521	0.516	0.139	0.182	0.013
rs242557	rs3785883	0.154	0.01	0.203	0.007
rs242557	rs2471738	0.661	0.136	0.563	0.113
rs242557	rs8070723	0.783	0.091	1.0	0.068
rs242557	rs7521	0.14	0.011	0.032	0.001
rs3785883	rs2471738	0.154	0.017	0.094	0.006
rs3785883	rs8070723	1.0	0.063	1.0	0.039
rs3785883	rs7521	0.053	0.001	0.189	0.02
rs2471738	rs8070723	1.0	0.046	1.0	0.024
rs2471738	rs7521	0.78	0.071	0.829	0.237
rs8070723	rs7521	1.0	0.271	0.87	0.163

**Table 5:** Association of A and G alleles in rs8070723 SNP with risk of PSP and PSP subtypes. ORs (95% CIs) were evaluated using logistic regression models, adjusted for sex and age, in the R statistical computing environment. ORs represent the effect per additional copy of the respective allele. Power values are reported for each OR, with significant effects consistently showing power >0.8. A Bonferroni correction for multiple testing (two association tests) established statistical significance at *p* ≤ 0.025.

	Allele Frequency (%) in rs8070723				
Allele	Patients with PSP (*n* = 73)	Controls (*n* = 93)	OR (95% CI)	OR Power	*p*-Value
A	89.0	75.3	2.620 [1.399–5.140]	0.90	0.0035
G	11.0	24.7	0.382 [0.195–0.715]	0.92	0.0035
	**PSP-RS Subtype (*n* = 46)**	**Controls** **(*n* = 93)**	**OR (95% CI)**	**OR Power**	***p*-Value**
A	91.3	75.3	4.004 [1.798–10.237]	0.95	0.0015
G	8.7	24.7	0.250 [0.098–0.556]	0.95	0.0015
	**vPSP Subtype (*n* = 27)**	**Controls** **(*n* = 93)**	**OR (95% CI)**	**OR Power**	***p*-Value**
A	85.2	75.3	1.516 [0.649–3.871]	0.49	0.3558
G	14.8	24.7	0.660 [0.258–1.541]	0.49	0.3558

Abbreviations: OR, odds ratio; CI, Confidence Interval; PSP, Progressive Supranuclear Palsy; RS, Richardson Syndrome PSP subtype; V, Variant PSP subtype.

**Table 6:** Association of *MAPT* haplotypes with risk of PSP. ORs (95% CIs) were derived from epiR package in the R statistical computing environment. ORs represent the effect per additional copy of the respective haplotype. Power values are reported for each OR, indicating the probability of correctly detecting a true effect; significant associations correspond to power >0.8. A Bonferroni correction for multiple testing (19 association tests) established statistical significance at *p* ≤ 0.0026.

	Haplotype Frequency (%)				
Haplotype	Patients with PSP (*n* = 73)	Controls (*n* = 93)	OR (95% CI)	OR Power	*p*-Value
H1b	21.9	15.0	1.584 [0.903–2.777]	0.36	0.1156
H2 *	10.1	23.4	0.370 [0.196–0.695]	0.90	0.0015
H1e	10.9	10.2	1.082 [0.535–2.186]	0.04	0.8585
H1d	12.2	7.3	1.728 [0.828–3.603]	0.31	0.1891
H1h	6.4	4.8	1.292 [0.499–3.342]	0.08	0.6318
H1c	4.4	5.2	0.754 [0.268–2.126]	0.08	0.7972
H1l	4.8	4.5	1.121 [0.397–3.165]	0.04	1.000
H1j **	1.3	6.2	0.201 [0.044–0.915]	0.67	0.0265
H1i	3.5	4.0	0.907 [0.282–2.918]	0.04	1.000
H1u	1.6	4.1	0.309 [0.065–1.478]	0.36	0.1951
H1m	2.7	2.1	1.282 [0.315–5.214]	0.05	0.7345
H1o	3.5	1.4	2.163 [0.508–9.204]	0.18	0.3068
H1x	2.5	1.9	1.282 [0.315–5.214]	0.05	0.7345
H1f	3.0	1.2	2.592 [0.468–14.349]	0.20	0.4108
H1z	2.9	1.1	2.592 [0.468–14.349]	0.20	0.4108
H1p	1.8	2.0	0.955 [0.210–4.333]	0.03	1.000
H1q	1.7	1.3	1.278 [0.178–9.181]	0.04	1.000
H1y [32]	0.6	1.5	0.421 [0.043–4.087]	0.12	0.6337
H1t **	2.0	0.2	3.881 [0.466–37.705]	0.39	0.0377

Abbreviations: OR, odds ratio; CI, Confidence Interval; PSP, Progressive Supranuclear Palsy. * *MAPT* haplotype with a *p* ≤ 0.0026 (Bonferroni corrected *p* cutoff). ** *MAPT* subhaplotype with a *p* ≤ 0.05 (nominal significant).

**Table 7:** Association of *MAPT* haplotypes with risk of PSP-RS. Statistical significance was defined as *p* ≤ 0.0027 after Bonferroni adjustment for 18 tests. ORs with power > 0.8 are considered reliably detectable and statistically robust.

	Haplotype Frequency (%)				
Haplotype	Patients with PSP-RS (*n* = 46)	Controls (*n* = 93)	OR (95% CI)	OR Power	*p*-Value
H2 *	7.5	23.3	0.274 [0.118–0.636]	0.95	0.0014
H1b	20.9	15.0	1.469 [0.770–2.800]	0.23	0.2400
H1e	13.8	10.1	1.446 [0.680–3.076]	0.17	0.4244
H1d	11.2	7.2	1.623 [0.683–3.855]	0.20	0.3543
H1h	8.8	5.2	1.676 [0.638–4.401]	0.19	0.3073
H1c	3.8	5.2	0.593 [0.159–2.210]	0.14	0.5548
H1j	1.7	6.2	0.322 [0.071–1.471]	0.42	0.1535
H1l	4.6	4.2	1.011 [0.296–3.450]	0.03	1.000
H1i	3.5	4.1	0.750 [0.194–2.896]	0.07	1.000
H1u	2.0	4.1	0.494 [0.103–2.377]	0.17	0.5050
H1m	3.6	2.1	1.534 [0.336–7.000]	0.08	0.6882
H1p	2.8	2.0	1.534 [0.336–7.000]	0.08	0.6882
H1q	2.5	1.4	1.356 [0.223–8.257]	0.05	0.6672
H1o	2.8	1.2	3.101 [0.509–18.89]	0.24	0.3362
H1x	1.1	1.9	0.500 [0.055–4.538]	0.10	1.000
H1y [32]	1.0	1.7	0.670 [0.069–6.535]	0.06	1.000
H1t	3.2	0.3	6.236 [0.640–60.80]	0.38	0.1074
H1f	1.7	0.9	2.044 [0.283–14.75]	0.11	0.6014

Abbreviations: OR, odds ratio; CI, Confidence Interval; PSP-RS, Progressive Supranuclear Palsy—Richardson Syndrome. * *MAPT* haplotype with a *p* ≤ 0.0027 (Bonferroni corrected *p* cutoff).

**Table 8:** Association of *MAPT* haplotypes with risk of vPSP. Bonferroni correction (18 tests) set significance at *p* ≤ 0.0027. ORs with power > 0.8 are considered statistically reliable.

	Haplotype Frequency (%)				
Haplotype	Patients with vPSP (*n* = 27)	Controls (*n* = 93)	OR (95% CI)	OR Power	*p*-Value
H2	14.5	23.4	0.561 [0.246–1.279]	0.41	0.1922
H1b	22.9	14.9	1.612 [0.756–3.437]	0.30	0.2183
H1e	5.9	10.5	0.488 [0.139–1.710]	0.31	0.3058
H1d	12.7	7.0	1.982 [0.749–5.248]	0.34	0.1686
H1c	5.7	5.5	1.035 [0.275–3.904]	0.03	1.000
H1j	1.0	6.3	0.274 [0.035–2.153]	0.43	0.3076
H1l	5.0	4.6	1.157 [0.302–4.433]	0.04	0.7350
H1i	5.3	4.3	1.309 [0.335–5.114]	0.07	0.7143
H1h	2.6	4.4	0.420 [0.051–3.433]	0.19	0.6881
H1u	1.1	4.0	0.482 [0.058–4.010]	0.14	0.6874
H1x	5.1	2.0	2.676 [0.580–12.35]	0.28	0.1909
H1o	4.7	1.5	3.588 [0.703–18.32]	0.37	0.1293
H1f	4.9	1.3	5.412 [0.880–33.26]	0.49	0.0768
H1z	5.3	1.0	5.412 [0.880–33.26]	0.49	0.0768
H1p	0.7	1.9	0.372 [0.020–7.019]	0.14	0.5773
H1m	0.3	1.8	0.481 [0.024–9.456]	0.09	0.5778
H1y [32]	0.1	1.5	0.481 [0.024–9.456]	0.09	0.5778
H2ff	0.3	1.3	0.677 [0.032–14.32]	0.05	1.000

Abbreviations: OR, odds ratio; CI, Confidence Interval; vPSP, Progressive Supranuclear Palsy Variant.

## Data Availability

The data that support the findings of this study are available from the corresponding author (R.P.) upon reasonable request.

## Supplementary Materials

The following supporting information can be downloaded at: https://www.mdpi.com/article/10.3390/biomedicines13061405/s1, Table S1: Allele frequency and association of rs8070723 with PSP and its subtypes; Table S2: Association of MAPT Haplotypes with risk of PSP; Table S3: MAPT H2 haplotypes and H1 sub-haplotypes that were observed in 1% or more of 46 PSP-RS cases and 93 HC in any of the 18 association analyses; Table S4: Association of MAPT Haplotypes with risk of PSP-RS; Table S5: MAPT H2 haplotypes and H1 sub-haplotypes that were observed in 1% or more of 27 vPSP cases and 93 HC in any of the 18 association analyses; Table S6: Association of MAPT Haplotypes with risk of vPSP.

## Abbreviations

The following abbreviations are used in this manuscript:

PSP	Progressive Supranuclear Palsy
PSP-RS	Progressive Supranuclear Palsy Richardson syndrome
vPSP	Progressive Supranuclear Palsy variants
PSP-P	Progressive Supranuclear Palsy with predominant parkinsonism
PSP-PGF	Progressive Supranuclear Palsy with progressive gait freezing
PSP-CBS	Progressive Supranuclear Palsy with predominant corticobasal syndrome
PSP-SL	Progressive Supranuclear Palsy with a predominant speech/language disorder
PSP-F	Progressive Supranuclear Palsy with predominant frontal presentation
PSP-C	Progressive Supranuclear Palsy with cerebellar ataxia
PSP-OM	Progressive Supranuclear Palsy with oculomotor dysfunction
NFTs	Neurofibrillary tangles
TA	Tufted astrocytes
CB	Oligodendroglial coiled bodies
NT	Neuropil threads
NTR	N-terminal region
PRR	Proline-rich domain
MTBD	Microtubule-binding domain
LD	Linkage disequilibrium
HC	Healthy control
r^2^	Squared correlation coefficient
EM	Expectation-Maximization algorithm
ORs	Odds ratios
CI	Confidence intervals
HWE	Hardy–Weinberg equilibrium
