# Supplementary material for: MAPT Subhaplotypes in Different Progressive Supranuclear Palsy Phenotypes
Source: Biomedicines. 2025 Jun 7;13(6):1405. doi: 10.3390/biomedicines13061405 (PMC12190731; doi:10.3390/biomedicines13061405)
Supplement: Supplementary file 1 [file biomedicines-13-01405-s001.zip › biomedicines-3660465-supplementary.pdf]

Supplementary Materials

Table S1. Allele frequency and association of rs8070723 with PSP and its subtypes. This table displays the allele frequencies (A and G) of rs8070723 among PSP patients (*N* = 73), PSP-RS subtype (*N* = 46), and vPSP subtype (*N* = 27) compared to controls (*N* = 93). It includes risk ratios (RR), 95% confidence intervals (CI), and *p*-values, highlighting a significant association of the A allele with PSP and PSP-RS.

	**Allele Frequency (%) in rs8070723**				
**Allele**	**Patients with PSP (*N* = 73)**	**Controls (*N* = 93)**	**Risk Ratio**	**CI (95%)**	***p*-value**
A	89.0	75.3	1.847	[1.269-2.485]	0.0004
G	11.0	24.7	0.544	[0.318-0.828]	0.0126

	**PSP-RS subtype (*N* = 46)**	**Controls (*N* = 93)**	**Risk Ratio**	**CI (95%)**	***p*-value**
A	91.3	75.3	2.771	[1.608-4.322]	0.0001
G	8.7	24.7	0.348	[0.148-0.667]	0.0061

	**vPSP subtype (*N* = 27)**	**Controls (*N* = 93)**	**Risk Ratio**	**CI (95%)**	***p*-value**
A	85.2	75.3	1.408	[0.684-2.716]	0.3354
G	14.8	24.7	0.720	[0.316-1.359]	0.3843

Table S2. Association of *MAPT* haplotypes with risk of PSP. For each haplotype, the risk ratio (RR), 95% confidence interval (CI), and the corresponding unadjusted and false discovery rate (FDR)-adjusted *p*-values are reported.

**Haplotype**	**Risk Ratio**	**CI (95%)**	***p*-value**	**adjusted *p*-value**
H1b	1.273	[0.942-1.592]	0.0716	0.3399
H2	0.530	[0.320-0.814]	0.0078	0.1478
H1e	1.044	[0.672-1.439]	0.8343	0.9288
H1d	1.318	[0.894-1.707]	0.0937	0.3561
H1h	1.146	[0.639-1.653]	0.5861	0.9288
H1c	0.846	[0.396-1.418]	0.6209	0.9288
H1l	1.064	[0.539-1.624]	0.8359	0.9288
H1j	0.315	[0.078-0.951]	0.0699	0.3399
H1i	0.946	[0.412-1.581]	0.8799	0.9288
H1u	0.447	[0.111-1.218]	0.1889	0.4486
H1m	1.141	[0.450-1.831]	0.7259	0.9288
H1o	1.436	[0.647-2.104]	0.2133	0.4504
H1x	1.141	[0.450-1.831]	0.7259	0.9288
H1f	1.531	[0.609-2.106]	0.1795	0.4486
H1z	1.531	[0.609-2.106]	0.1795	0.4486
H1p	0.974	[0.322-1.757]	0.9559	0.9559
H1q	1.139	[0.278-2.0]	0.8080	0.9288
H1y	0.566	[0.075-1.728]	0.4859	0.9232
H1t	2.013	[0.607-2.284]	0.0382	0.3399

Table S3. *MAPT* H2 haplotypes and H1 sub-haplotypes that were observed in 1% or more of 46 PSP-RS cases and 93 HC in any of the 18 association analyses.

	***MAPT* variant**					
**Haplotype**	**rs1467967**	**rs242557**	**rs3785883**	**rs2471738**	**rs8070723**	**rs7521**
H2	A	G	G	C	G	G
H1b	G	G	G	C	A	A
H1e	A	G	G	C	A	A
H1d	A	A	G	C	A	A
H1h	A	G	A	C	A	A
H1c	A	A	G	T	A	G
H1j	A	G	G	C	A	G
H1l	A	G	A	C	A	G
H1i	G	A	G	C	A	A
H1u	A	A	G	C	A	G
H1m	G	A	G	C	A	G
H1p	G	G	G	T	A	G
H1q	A	A	G	T	A	A
H1o	A	A	A	C	A	A
H1x	G	A	A	T	A	G
H1y [32]	G	A	A	C	A	G
H1t	A	G	A	T	A	G
H1f	G	G	A	C	A	A

Table S4. Association of *MAPT* haplotypes with risk of PSP-RS.

**Haplotype**	**Risk Ratio**	**CI (95%)**	***p*-value**	**adjusted *p*-value**
H2	0.376	[0.176-0.736]	0.0074	0.1327
H1b	1.279	[0.831-1.785]	0.2085	0.6248
H1e	1.265	[0.758-1.846]	0.3046	0.6853
H1d	1.352	[0.761-2.010]	0.2257	0.6248
H1h	1.376	[0.723-2.097]	0.2430	0.6248
H1c	0.687	[0.222-1.571]	0.4613	0.7084
H1j	0.419	[0.103-1.267]	0.1747	0.6248
H1l	1.008	[0.386-1.906]	0.9867	0.9867
H1i	0.818	[0.266-1.775]	0.6919	0.7794
H1u	0.596	[0.147-1.625]	0.4049	0.7084
H1m	1.305	[0.430-2.357]	0.5510	0.7084
H1p	1.305	[0.430-2.357]	0.5510	0.7084
H1q	1.213	[0.299-2.434]	0.7307	0.7794
H1o	1.840	[0.606-2.765]	0.1151	0.6248
H1x	0.600	[0.080-2.082]	0.5495	0.7084
H1y	0.753	[0.100-2.302]	0.7361	0.7794
H1t	2.309	[0.724-2.977]	0.0201	0.1812
H1f	1.522	[0.371-2.674]	0.4120	0.7084

Table S5. *MAPT* H2 haplotypes and H1 sub-haplotypes that were observed in 1% or more of 27 vPSP cases and 93 HC in any of the 18 association analyses.

	***MAPT* variant**					
**Haplotype**	**rs1467967**	**rs242557**	**rs3785883**	**rs2471738**	**rs8070723**	**rs7521**
H2	A	G	G	C	G	G
H1b	G	G	G	C	A	A
H1e	A	G	G	C	A	A
H1d	A	A	G	C	A	A
H1c	A	A	G	T	A	G
H1j	A	G	G	C	A	G
H1l	A	G	A	C	A	G
H1i	G	A	G	C	A	A
H1h	A	G	A	C	A	A
H1u	A	A	G	C	A	G
H1x	G	A	A	T	A	G
H1o	A	A	A	C	A	A
H1f	G	G	A	C	A	A
H1z	G	A	G	T	A	G
H1p	G	G	G	T	A	G
H1m	G	A	G	C	A	G
H1y [32]	G	A	A	C	A	G
H2ff	A	A	G	C	G	G

Table S6. Association of *MAPT* haplotypes with risk of vPSP.

Haplotype	Risk Ratio	CI (95%)	*p*-value	adjusted *p*-value
H2	0.629	[0.302-1.197]	0.1877	0.4827
H1b	1.429	[0.797-2.274]	0.1833	0.4827
H1e	0.555	[0.175-1.465]	0.2813	0.5626
H1d	1.638	[0.791-2.751]	0.1205	0.4473
H1c	1.027	[0.328-2.363]	0.9615	0.9615
H1j	0.329	[0.045-1.696]	0.2331	0.5244
H1l	1.118	[0.358-2.507]	0.8341	0.8831
H1i	1.225	[0.393-2.669]	0.6915	0.8298
H1h	0.484	[0.066-2.203]	0.4265	0.7587
H1u	0.547	[0.074-2.376]	0.5058	0.7587
H1x	1.958	[0.639-3.544]	0.1242	0.4473
H1o	2.294	[0.752-3.837]	0.0455	0.2729
H1f	2.765	[0.904-4.157]	0.0090	0.0808
H1z	2.765	[0.904-4.157]	0.0090	0.0808
H1p	0.434	[0.025-2.953]	0.5020	0.7587
H1m	0.546	[0.031-3.231]	0.6205	0.7978
H1y	0.546	[0.031-3.231]	0.6205	0.7978
H2ff	0.731	[0.041-3.560]	0.7951	0.8831
